# Choropleth Map Design for Cancer Incidence, Part 1

**Published:** 2009-12-15

**Authors:** Thomas B. Richards, Zahava Berkowitz, Cheryll C. Thomas, Stephanie Lee Foster, Annette Gardner, Jessica Blythe King, Karen Ledford, Janet Royalty

**Affiliations:** Centers for Disease Control and Prevention; Centers for Disease Control and Prevention, Atlanta, Georgia; Centers for Disease Control and Prevention, Atlanta, Georgia; Centers for Disease Control and Prevention, Atlanta, Georgia. Stephanie Lee Foster is also affiliated with the Agency for Toxic Substances and Disease Registry, Atlanta, Georgia; Centers for Disease Control and Prevention, Atlanta, Georgia; Centers for Disease Control and Prevention, Atlanta, Georgia; Centers for Disease Control and Prevention, Atlanta, Georgia; Centers for Disease Control and Prevention, Atlanta, Georgia

## Abstract

Choropleth maps are commonly used in cancer reports and community discussions about cancer rates. Cancer registries increasingly use geographic information system techniques. The Centers for Disease Control and Prevention's Division of Cancer Prevention and Control convened a Map Work Group to help guide application of geographic information systems mapping techniques and to promote choropleth mapping of data from central cancer registries supported by the National Program of Cancer Registries, especially for planning and evaluation of comprehensive cancer control programs. In this 2-part series in this issue of *Preventing Chronic Disease*, we answer frequently asked questions about choropleth map design to display cancer incidence data. We recommend that future initiatives consider more advanced mapping, spatial analysis, and spatial statistics techniques, and include usability testing with representatives of state and local programs and other cancer prevention partners.

## Introduction

Maps are an effective tool for cancer control planning and evaluation (1-3). Data displayed on a map allow users to visualize spatial relationships and draw attention to areas of importance. Maps can be used to identify boundaries of complex geography, display rates for specific areas, reveal geographic patterns, and suggest questions for research (eg, what is the spatial relationship between cancer rates and risk factors such as socioeconomic status?) (1).

The National Program of Cancer Registries (NPCR), Division of Cancer Prevention and Control (DCPC), Centers for Disease Control and Prevention (CDC) supports state central cancer registries (CCR) in the collection of high-quality cancer incidence data (4). An increasing number of these registries assign geocodes (eg, latitude and longitude coordinates) to residential addresses of people with incident cases (5,6). These geocoded cases can be used to develop maps of cancer incidence rates and as part of spatial statistical analyses (7).

Choropleth maps are a common starting point for mapping cancer incidence. DCPC convened a Map Work Group to develop guidance for the design of choropleth maps and to promote mapping of NPCR-supported CCR cancer incidence data. Choropleth maps of cancer incidence rates assign colors to rate categories and then fill the area in the geographic units of interest (eg, states, counties, census tracts) with the color corresponding to that unit's rate (8). The National Cancer Institute (NCI) and CDC state cancer profiles Web site provides good examples of choropleth maps (9). Many more advanced mapping methods exist, but these methods typically require investment in additional software or training for state program staff (7,10,11).

This 2-part series summarizes Map Work Group responses to common questions about choropleth map design. In this article we discuss the purpose of the map, geographic units of analysis, cancer sites, age-adjusted rates, rate ratios, and reliability. In Part 2 we discuss suppression rules to protect the privacy and confidentiality of cancer patients; questions related to mapping cancer stages, rates, and percentages; classes for map display; comparing maps over time; map color schemes, labels, projections, and output media; and limitations in interpretation (12).

## Frequently Asked Questions About Choropleth Map Design

### 1. *What is the purpose of the map?*

Map design requires consideration of the audiences to which the map will be presented, the purpose that the map serves for each audience, and plans to provide supplemental information to help interpret the map. For example, in the context of comprehensive cancer control, multiple audiences potentially exist, including community members, policy makers, clinicians, geographers, epidemiologists, and state comprehensive cancer control staff. For internal program use by CCR staffers who have signed an agreement to protect privacy and confidentiality of cancer data, maps can be useful to show point locations of cancer cases. However, to protect privacy and confidentiality, this type of map would not be distributed to the public. Similarly, although maps developed with advanced GIS methods (eg, adaptive spatial filtering) can be used to engage community participation (11), such maps may require that the map maker meet with community representatives to explain the methods used and how to interpret the map.

Sharing maps with end users during development ensures that the content, meaning, and audience interpretation are appropriate. More formal usability testing may be helpful, especially when requesting user feedback on Web applications with maps (13). The same map may not be equally suited for all audiences or be able to answer all questions. A single map may lead end users to request additional maps. More than 1 map or different types of maps in addition to tables, graphs, and explanatory text may be needed to answer all of the questions posed by a specific audience.

Maps are especially useful to help users visualize the answers to "where" questions and questions about spatial relationships. Such questions are commonly asked as part of state comprehensive cancer control planning and evaluation (1), for example:

- Where are high-priority populations for cancer prevention interventions?
- Where are cancer screening services provided?
- Where do preventable cancers occur, especially advanced-stage cases?
- Are there gaps between the locations of high-priority populations and locations where cancer prevention services are provided?
- What is the cancer incidence rate for a specific area?
- Where are areas with unusually high or low rates?
- Are the geographic patterns on a map caused by normal random variation?
- How do spatial patterns in cancer incidence rates change over time?
- What is the spatial correlation between geographic patterns for cancer incidence rates and those for cancer risk factors?

### 2. *Are some geographic units of analysis more advantageous than others for choropleth cancer incidence maps?*

In 2003, Boscoe and Pickle (14) reviewed 12 geographic units that can be used for choropleth maps of cancer incidence data and identified the following characteristics as desirable:

- high degree of resolution
- homogeneity of population size
- homogeneity of land area
- observation of minimum population thresholds and land area thresholds
- temporal stability and currency
- compactness of shape
- audience familiarity
- data availability
- the functional relevance of the unit to the phenomena mapped

They concluded that 1) each of the 12 geographic units had some advantages and disadvantages; 2) depending on the specific study question, some units may be preferable to others; and 3) none of the units was optimal for all purposes (14). For national maps of the continental United States, they assigned highest ratings to states, counties, and the Health Service Areas used in CDC's *Atlas of United States Mortality* (14,15).

In addition to the units reviewed by Boscoe and Pickle (14), Hao et al (16) suggest that presentation of cancer data using congressional district boundaries may be useful in communicating with legislators and persuading them to enact new cancer control programs and to strengthen existing ones. Because many congressional districts do not follow state or county boundaries, Hao et al (16) describe a method to estimate age-adjusted death rates for congressional districts by using county-level data.

Other investigators have concluded that analyses using geographic units at the subcounty level would be advantageous. For example, Goodman et al (17) define primary care service areas based on US zip codes where Medicare beneficiaries prefer to receive primary care. California has mapped advanced-stage colon cancer cases by using medical service study areas, based on aggregations of census tracts that local communities considered "rational service areas" for primary health care (18). Gregorio et al (19) suggest that, except for investigations focused on a specific cancer cluster in a limited geographic area, spatial analysis at the census tract level might be a sufficient resolution for surveillance of cancer spatial patterns in a single state.

An additional consideration in choice of geographic unit may be the ability to use geography to accurately link cancer incidence data with census demographics, risk factors, and other data. In 2002, Krieger et al (20) concluded that census tract or block group units were better than zip codes for analyses of US socioeconomic inequalities in health.

### 3. *What cancer sites would be good starting points for illustrating how cancer registry data may be used to help answer cancer prevention and control questions?*

The Map Work Group recommended breast, colorectal, and cervical cancer as reasonable starting points for the development of cancer incidence maps for comprehensive cancer control. These cancers can be prevented by implementation of the US Preventive Services Task Force (USPSTF) recommendations for community preventive services and clinical interventions (21). The USPSTF recommends screening men and women aged 50 years or older for colorectal cancer; biennial screening mammography for women aged 50 to 74 years; and screening for cervical cancer in women who have been sexually active and have a cervix.

Other cancer sites and types of data also may be of interest. For example:

- For a specific state, any high-priority cancer identified in the state comprehensive cancer control plan (22).
- For lung cancer, maps and geographic analyses of trends in tobacco use by high school students (23).

### 4. *What types of questions are best addressed by* *maps showing incident cancer case counts, unadjusted (crude) rates, direct age-adjusted rates, or indirect age-adjusted rates?*

Presenting cancer case counts in a table with the geographic unit (eg, county) as the row can be a useful starting point for cancer prevention and control discussions. On the basis of information in the table, a choropleth map of case counts can be developed. However, because case counts are often proportional to population size, decision makers may ask questions that require tables showing the case-to-population ratio or rate by geographic unit and choropleth maps designed on the basis of that information.

Rates for many cancers increase with age, and differences in the population age distribution in different areas can influence the observed crude cancer rates in each area. To control for such differences, direct and indirect methods can be used for age adjustment (sometimes referred to as age standardization) (11,24,25).

The direct age-adjusted rate is calculated by multiplying the age-specific crude rates for the local study population (eg, for a county) by the corresponding age-specific proportion weight for the standard population (eg, for a state) and then summing these products. Direct age-adjusted rates are reported in the NCI/CDC *State Cancer Profiles* and in *United States Cancer Statistics* reports, using the national population as the standard (9,26)*.*

In contrast, the indirect age-adjusted method estimates the expected cases in the local study area (eg, a county) by multiplying the number of people in an age category for the local study area population by the corresponding age-specific rates of the standard population (eg, the state). Expected cases then are summed across age groups and compared with the actual or observed number of cases in the local study population. The ratio of observed to expected cases takes into account age distribution because both the observed and expected cases are based on the age distribution of the local study population.

If the goal is to compare cancer rates in different local study populations, direct age-adjusted rates are needed. Indirect age-adjustment does not allow rates in different local study populations to be compared because indirect age-adjusted rates are not based on a common age distribution. However, the indirect age-adjustment method can be advantageous when the local study population age groups are too small to calculate stable, local age-specific rates, as in sparsely populated rural counties (11). As summarized by Beyer and Rushton, indirect age-adjustment "applies the stable statewide rate to local populations, instead of applying local disease rates, which for small areas are unstable, to standard population weights" (11).

For questions about allocation of resources, tables of case counts and maps of age-specific rates may be more useful than age-adjusted rates because the case counts and age-specific rates are actual measures of risk within the specific area of interest. In contrast, direct age-adjusted rates are relative indexes, and hypothetical rates reflect the age distribution of the selected standard population rather than the actual number of people in each age category in a specific community. A potential limitation of indirect age-adjusted rates for purposes of resource allocation is that each local area applies a different set of weights reflecting the age distribution of its population. On the other hand, as Beyer and Rushton point out, local decision makers may find indirect age-adjusted rates useful because "the difference between actual and expected numbers of late-stage cancer cases is a measure of the need for additional resources such as screening services" (11).

### 5. *When calculating and evaluating county-to-state rate ratios to identify specific counties with higher or lower rates than the state rate, how should the denominator for the rate ratio be defined when the index county of interest has a relatively large population compared with other counties in that state?*

In 2008, 16 counties (in 15 states) accounted for 25% or more of the total population in their state. Examples of such counties are Clark County, Nevada (71.8% of the state population); Maricopa County, Arizona (60.8%); Cook County, Illinois (41.0%); Salt Lake County, Utah (37.4%); King County, Washington (28.6%); and Los Angeles County, California (26.8%) (27).

County-to-state rate ratios are calculated as the ratio of the rate for an index county of interest to the state rate. The state rate can potentially be calculated with the index county excluded (State Rate 1) or with the index county included (State Rate 2).

State Rate 1 (excluding the index county) is advantageous from a statistical perspective because the numerator rate (the index county rate) and denominator rate (the state rate excluding the index county) are independent. The statistical assumption of nonoverlapping groups is not violated.

State Rate 2 (including the index county) allows overlap between the numerator rate (the index county rate) and the denominator rate (the state rate including the index county). However, if county-to-state rate ratios are needed for every county in a state, the State Rate 2 approach is easier to calculate than the State Rate 1 approach. Using the State Rate 2 approach, the rate for each index county is compared with the same denominator (the state rate including the index county). In contrast, using the State Rate 1 (excluding the index county) approach, a different state rate needs to be calculated with the selection of each index county.

The Map Work Group concluded that the following rule of thumb may be helpful in deciding which approach would be appropriate. The state rate can be calculated with the index county included (State Rate 2) if the population of the index county accounts for less than 25% of total state population. However, if the population of the index county accounts for 25% or more of the total state population, then the state rate should exclude the index county (State Rate 1). When an index county accounts for 25% or more of the total state population, inclusion of the index county in the state rate can result in a reported county-to-state rate ratio that is less than the true county-to-state rate ratio by at least 10%.

### 6. *How should information about reliability (eg, unstable rates) be displayed on a map?*

Several methods exist to display information about reliability of rates on a map. One option uses different shades of gray to indicate map areas with small numbers, unstable rates, or missing data. If colors are used to indicate areas with stable rates, areas shaded gray tend to remain in the background.

A second option employs hatched lines to convey rate variance. The hatched lines allow the underlying spatial patterns to be seen. The *Atlas of United States Mortality* (15) illustrates how double-hatching with parallel white and black lines can be used over light and dark colors.

A third method, proposed by Carr and colleagues (28,29), provides confidence intervals in addition to mapped rates. The mapped rates are ranked, confidence intervals are calculated around each rate, and a graph of the ranked rates with their confidence intervals is then displayed adjacent to micromaps of the rates. This approach is used in the Comparative Data Display section of the *State Cancer Profiles* (9).

A fourth option is the use of funnel plots. Funnel plots show increasing population size on the x-axis, and higher and lower bounds for predicted limits for rates on the y-axis around a horizontal line corresponding to the overall rate (30,31). The predicted limits decrease as population size increases, resulting in a graph with a shape similar to that of a funnel. Outliers for geographic units of different population sizes are identifiable as the rates located outside the predicted limits.

## Conclusion

Design of high-quality, effective choropleth maps of cancer incidence may appear simple but in fact can involve complex issues.
